# Association between decision-to-delivery time and neonatal outcomes: a systematic review and meta-analysis

**DOI:** 10.1186/s12884-024-06603-y

**Published:** 2024-06-07

**Authors:** Jiali Shen, Minmin Shi

**Affiliations:** Department of Emergency, Huzhou Maternity & Child Health Care Hospital, 2 East Street, Wuxing District, Huzhou, Zhejiang Province China

**Keywords:** APGAR, Decision to delivery time, Meta-analysis, Neonates

## Abstract

**Background:**

Decision-to-delivery time (DDT), a crucial factor during the emergency caesarean section, may potentially impact neonatal outcomes. This study aims to assess the association between DDT and various neonatal outcomes.

**Methods:**

A comprehensive search of PubMed, Scopus, Cochrane Library, and Google Scholar databases was conducted. A total of 32 eligible studies that reported on various neonatal outcomes, such as Apgar score, acidosis, neonatal intensive unit (NICU) admissions and mortality were included in the review. Studies were selected based on predefined eligibility criteria, and a random-effects inverse-variance model with DerSimonian-Laird estimate of tau² was used for meta-analysis. Heterogeneity and publication bias were assessed using I² statistics and Egger’s test, respectively.

**Results:**

The meta-analysis revealed a significant association between DDT < 30 min and increased risk of Apgar score < 7 (OR 1.803, 95% CI: 1.284–2.533) and umbilical cord pH < 7.1 (OR 4.322, 95% CI: 2.302–8.115), with substantial heterogeneity. No significant association was found between DDT and NICU admission (OR 0.982, 95% CI: 0.767–1.258) or neonatal mortality (OR 0.983, 95% CI: 0.565–1.708), with negligible heterogeneity. Publication bias was not detected for any outcomes.

**Conclusions:**

This study underscores the association between shorter DDT and increased odds of adverse neonatal outcomes such as low Apgar scores and acidosis, while no significant association was found in terms of NICU admissions or neonatal mortality. Our findings highlight the complexity of DDT’s impact, suggesting the need for nuanced clinical decision-making in cases of emergency caesarean sections.

## Introduction

An emergency caesarean section is often necessary in cases when there is an immediate threat to the life of a mother and a fetus. In such instances, a delay in delivery may have profound implications on neonatal outcomes [1]. The period between a decision to perform the emergency caesarean section and the actual delivery of the neonate is called decision to delivery time (DDT) and should not exceed 30 min [2, 3]. The World Health Organization (WHO) also underscores the importance of a DDT < 30 for emergency caesarean deliveries [4]. However, their recommendations are based on expert consensus rather than robust empirical evidence. Moreover, there are varying interpretations and implementations of these guidelines across different health systems and regions [5–7].

In recent years, there has been an increase in research focusing on the relationship between DDT and the actual neonatal outcomes [8–10]. However, the results of these studies are inconsistent and sometimes contradictory [8–10]. While some reports have found a significant association between prolonged DDT and adverse neonatal outcomes [11], others have reported minimal or no impact.^8–10^ These inconsistencies could be attributed to varying study designs, differences in healthcare settings, and diverse patient populations.

Neonatal outcomes, such as mortality, low Apgar scores, acidosis as indicated by umbilical artery pH, and the need for admission to the Neonatal Intensive Care Unit (NICU) serve as critical markers of the quality of perinatal care [12]. These outcomes are intricately linked to the timing of delivery in emergent situations, making them ideal parameters to evaluate the implications of DDT. This study aims to synthesize the evidence on the association between a DDT of greater than 30 min versus less than 30 min and key neonatal outcomes, namely neonatal mortality, reduced Apgar score, reduced umbilical artery pH, and NICU admission.

## Methods

The study protocol was registered with the International Prospective Register of Systematic Reviews (PROSPERO). **Registration Number**: CRD42023489315.

### Eligibility criteria

#### Study characteristics

We included studies that investigated the association between DDT and neonatal outcomes (neonatal mortality, Apgar score < 7, umbilical pH < 7.1, NICU admission). Observational study design such as cohort (prospective/retrospective), case-control, cross-sectional were considered.

#### Participants

Studies involving pregnant women undergoing emergency caesarean section.

#### Interventions/Comparators

The primary comparison was between DDT > 30 min vs. <30 min.

#### Outcomes

Neonatal mortality, Apgar score < 7, umbilical artery pH < 7.1, and NICU admission.

#### Settings

There were no restrictions on the setting or location of the studies.

#### Language and publication status

Studies published in English in peer-reviewed journals were included. Unpublished studies, conference abstracts, and grey literature were excluded.

### Information sources and search strategy

We searched the following databases: PubMed, Scopus, Cochrane Library, and Google Scholar for literature published from inception of these databases to November 2023.

The search strategy was developed with the assistance of a medical librarian and included a combination of keywords and MeSH terms related to “decision-to-delivery time,” “emergency cesarean section,” and “neonatal outcomes.” Model search strategy is as follows: (“decision-to-delivery time” OR “emergency cesarean section”) AND (“neonatal outcomes” OR “neonatal mortality” OR “Apgar score” OR “umbilical pH” OR “NICU admission”).

Additional studies were identified by scanning reference lists of included studies and relevant reviews. Experts in the field were also consulted for unpublished or ongoing studies.

### Study selection and data collection

Titles and abstracts of identified studies were screened independently by both the authors for potential inclusion using Rayyan online software. Full texts of potentially relevant studies were then independently assessed for eligibility. Discrepancies were resolved through discussion between them. A PRISMA flow diagram was used to document the process of study selection [13]. A standardized form was used for data extraction. Both the authors independently extracted data, including study design, sample size, participant characteristics, details of the exposure and comparator groups, outcome measures, and study findings. Discrepancies in data extraction were resolved through discussion between two reviewers.

Primary and secondary outcomes were clearly defined, with primary outcomes given precedence in the analysis.

### Quality assessment

The quality of individual studies was assessed using the Newcastle-Ottawa Scale [14] for observational studies, which consists of selection, comparability and outcome domains. Findings on the quality assessment were systematically documented and considered in the interpretation of the review’s results as low, moderate and high quality.

### Statistical analysis

In our analysis, we employed the DerSimonian and Laird random effects model with inverse variance approach.^14^ To address the outcomes of interest in our study, we meticulously extracted data to construct 2 × 2 contingency tables for each included study. These tables detailed the number of participants exposed and not exposed to the risk factor, alongside those who did and did not experience the outcome of interest. Utilizing this approach allowed for a comprehensive and precise analysis of the relationship between exposure and outcome across the studies.

For the pooling of findings from these contingency tables, we employed the *‘metan’* package, a robust tool designed for meta-analysis in statistical software. This package facilitated the accurate calculation and pooling of odds ratios from the individual studies, providing a consolidated measure of the effect size.

The decision to use odds ratios as our primary measure of association was informed by the nature of the included studies in our analysis. Given that the majority of these studies were retrospective or cross-sectional in design, with only a few being prospective, odds ratios emerged as the most appropriate and informative measure. Odds ratios are particularly suited for this study design mix, as they offer a reliable estimate of the risk associated with the exposure, even when the absolute risk is not known. This measure effectively captures the strength of the association between the exposure and the outcome across a variety of study designs, ensuring the relevance and applicability of our findings.

The final effect size was reported as pooled odds ratio (OR) with 95% confidence interval (CI). Forest plot was used for visual representation of the overall results for each of the outcomes. *P* < 0.05 indicated significant association.

Heterogeneity among studies was assessed using the I² statistic. In our analysis, alongside the I-squared statistic, we utilized Tau-square and prediction intervals to assess heterogeneity among the included studies. Tau-square offers an estimate of the between-study variance, providing a more nuanced understanding of heterogeneity, while prediction intervals give a range in which we expect the true effects to lie in similar future studies. Methods for assessing reporting biases, such as publication bias, included the use of funnel plots and Egger’s regression test. *P* < 0.05 on Egger’s test indicates statistically significant publication bias [15]. Sensitivity analysis was performed for the assessment of heterogeneity. All the analysis was performed using STATA 17 software.

We applied the Grading of Recommendations Assessment, Development and Evaluation (GRADE) approach to assess the quality of evidence for each of the outcomes. The GRADE framework facilitated a systematic evaluation of the evidence, considering factors such as study limitations, inconsistency, indirectness, imprecision, and publication bias. This approach enabled us to categorize the evidence into four levels: high, moderate, low, or very low.

## Results

**Fig. 1:**
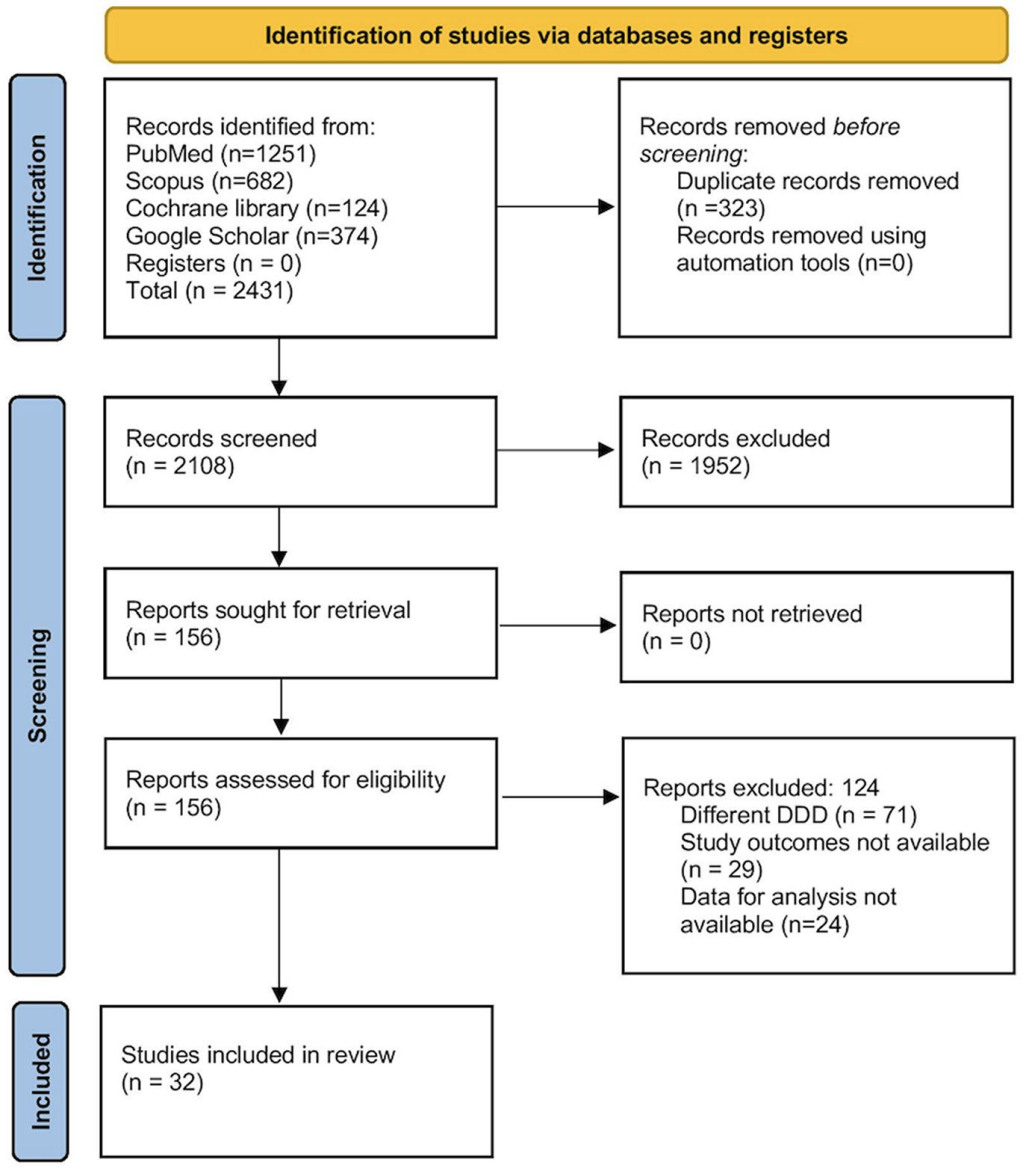
PRISMA flowchart

A total of 2431 records were identified during the initial search. After duplicate removal and primary screening, 156 full texts were obtained. Eventually, 32 eligible studies were included in review and analysis (Fig. 1) [8–10, 16–44].

### Characteristics of the included studies

The included studies were predominantly cross-sectional and prospective in nature, with a substantial representation from the USA, Ethiopia, and India. The sample sizes varied widely, ranging from 68 to 39,291 participants per study. The level of care was predominantly tertiary (level 3), reflecting the critical nature of cases (Table 1). Regarding the quality assessment, studies were diverse in quality: 3 studies were rated as having low quality, 20 with a moderate quality, and 9 with high quality (Tables 2 and 3).

**Table 1:** Meta-analysis Baseline

Study Identifier	Study design	Country	Study participants	Sample size	Level of care	Mean age (in years)	Category as per Lucas	Level of urgency	Risk of bias grade^#^ (score)
Anuradha 2020	Cross sectional study	India	Women who underwent emergency caesarean section	409	3	NR	1 and 2	Emergency	High (3)
Ayele 2021	Cross sectional study	Ethiopia	Inpatients undergoing emergency caesarean section	510	3	27.1	NR	Emergency	High (3)
Bello 2015	Prospective	Nigeria	Women who underwent emergency caesarean section	235	3	29.7	NR	Emergency	7 (Low)
Bloom 2006	Prospective	USA	Women who underwent primary caesarean delivery	2808	3	NR	NR	Emergency	6 (Moderate)
Boriboonhirunsarn 2016	Cross sectional study	Thailand	Term, singleton pregnant women who underwent an emergency caesarean section	272	3	28.7	NR	Emergency	5 (Moderate)
Bousleiman 2020	Retrospective	USA	Women 37 weeks of gestational age at delivery with no more than one prior caesarean and currently undergoing emergency caesarean section	5784	1,2,3	NR	NR	Emergency	5 (Moderate)
Chauhan 1997	Retrospective	USA	Women who underwent emergency caesarean section	117	3	NR	1,2	Emergency	5 (Moderate)
Chauhan 2000	Retrospective	USA	Women who underwent emergency caesarean section	84	3	NR	1,2	Emergency and urgent	5 (Moderate)
Chauleur 2009	Prospective	France	Women who have given birth with non-prophylactic caesarean section	68	3	NR	1,2	Overall, Class 1 and 2	5 (Moderate)
Dorjey 2023	Retrospective	Bhutan	Mothers who underwent Category-I emergency caesarean section	78	3	NR	1	Emergency	3 (High)
Grobman 2018	Prospective	USA	Women with a term, singleton, cephalic non-anomalous gestation and no prior caesarean delivery, who underwent an intrapartum caesarean delivery	3482	1,2,3	NR	NR	Emergency	6 (Moderate)
Heller 2017	Prospective	Germany	Women with in-hospital caesarean sections	39,291	1,2,3	NR	NR	Emergency	4 (Moderate)
Hillemmanns 2003	Retrospective cohort	Germany	Women undergoing ‘crash’ emergency caesarean section and controls	208	3	30.6	NR	Emergency	9 (Low)
Hirani 2017	Cross-sectional study	Tanzania	Women who underwent emergency caesarean section	598	3	29.2	1,2	Emergency	4 (Moderate)
Holcroft 2005	Retrospective cohort	USA	Women who underwent emergency caesarean section	117	3	NR	1,2	Emergency and urgent	6 (Moderate)
Huissoud 2010	Prospective observational	France	Women who underwent emergency caesarean section	447	1,2,3	29.5	1,2	Very urgent and urgent	6 (Moderate)
Khemworapong 2018	Retrospective cohort	Thailand	Women who underwent emergency caesarean section	431	3	30	NR	Emergency	5 (Moderate)
Kitaw 2021	Prospective cohort	Ethiopia	Women who underwent emergency caesarean section	182	3	27	NR	Emergency	7 (Low)
Kolas 2006	Prospective	Norway	Women who underwent urgent, emergency and acute caesarean section	1511	2,3	29.3	1	Urgent, emergency and acute	4 (Moderate)
Lavery 1999	Retrospective	USA	Women who underwent non-elective caesarean section	378	3	NR	1,2	Non-elective	5 (Moderate)
MacKenzie 2002	Prospective	United Kingdom	Women who underwent crash and emergency caesarean section	352	3	NR	1,2	Crash and emergent	5 (Moderate)
Mishra 2018	Prospective	India	Women with immediate threat or maternal or foetal compromise	480	3	NR	1,2	Emergency	3 (Low)
Nakintu 2016	Cross-sectional study	Uganda	Women who underwent emergency caesarean section	297	3	NR	1,2	Emergency	6 (Moderate)
Nasrallah 2004	Retrospective	USA	Women who underwent emergency caesarean section	111	3	NR	1,2	Emergency	5 (Moderate)
Pearson 2011	Prospective	United Kingdom	Women who underwent emergency caesarean section	546	3	NR	1	Category 1 & 2	6 (Moderate)
Schauberger 1994	Retrospective	USA	Women who underwent emergency caesarean section	75	3	NR	1,2	Emergency	6 (Moderate)
Singh 2012	Prospective	India	Women who underwent emergency caesarean section	204	3	26.5	NR	Emergency	7 (Low)
Sunsaneevithayakul 2022	Retrospective	Thailand	Women who underwent emergency caesarean section	254	3	29.8	3	Emergency	3 (High)
Tashfeen 2017	Cross-sectional study	Oman	Women with singleton pregnancies delivered by emergency caesarean section due to fetal distress, antepartum hemorrhage or umblical cord prolapse	246	3	NR	NR	Emergency	3 (High)
Temesgen 2020	Prospective	Ethiopia	Women who underwent category 1 emergency caesarean section	163	3	NR	1	Category I emergency	7 (Low)
Thomas 2004	Cross-sectional study	England and Wales	Women who underwent emergency caesarean section	17,780	3	NR	NR	Urgent	2 (High)
Tuffnell 2001	Prospective	United Kingdom	Women who underwent emergency caesarean section	721	3	NR	1,2	Urgent and emergent	6 (Moderate)

NR – Not reported; USA – United States of America;

^**#**^Risk of bias score: 0–3 = high risk; 4–6 = moderate risk and 7–9 = low risk

**Table 2:** Quality assessment of cohort studies

Study Identifier	Representativeness of exposed cohort	Selection of non-exposed cohort	Ascertainment of exposure	Outcome not present at start	Comparability of cohorts	Outcome assessment	Follow-up long enough	Adequacy of cohort follow-up	Quality points^#^ (Grade)
Bello 2015	0 point	1 point	1 point	1 point	2 points	1 point	0 point	1 point	7 (High quality)
Bloom 2006	1 point	1 point	1 point	1 point	1 point	1 point	0 point	0 point	6 (Moderate)
Bousleiman 2020	0 point	1 point	1 point	0 point	1 point	1 point	0 point	1 point	5 (Moderate)
Chauhan 1997	0 point	1 point	1 point	1 point	0 points	1 point	0 point	1 point	5 (Moderate)
Chauhan 2000	0 point	1 point	1 point	1 point	1 point	1 point	0 point	0 point	5 (Moderate)
Chauleur 2009	1 point	0 point	1 point	1 point	1 points	1 point	0 point	0 point	5 (Moderate)
Dorjey 2023	0 point	0 point	0 point	1 point	1 point	1 point	0 point	0 point	3 (Low)
Grobman 2018	1 point	1 point	1 point	1 point	2 points	1 point	0 point	0 point	6 (Moderate)
Heller 2017	1 point	1 point	1 point	1 point	2 points	0 point	0 point	1 point	6 (Moderate)
Hillemmanns 2003	1 point	1 point	1 point	1 point	2 points	1 point	1 point	1 point	9 (High)
Holcroft 2005	1 point	0 point	0 point	1 point	2 points	1 point	0 point	1 point	6 (Moderate)
Huissoud 2010	1 point	1 point	1 point	1 point	0 points	1 point	0 point	1 point	6 (Moderate)
Khemworapong 2018	1 point	0 point	1 point	0 point	1 point	1 point	0 point	1 point	5 (Moderate)
Kitaw 2021	1 point	1 point	1 point	1 point	2 points	1 point	0 point	0 point	7 (High)
Kolas 2006	0 point	1 point	0 point	1 point	0 points	1 point	0 point	1 point	4 (Moderate)
Lavery 1999	0 point	0 point	1 point	1 point	1 point	1 point	0 point	1 point	5 (Moderate)
MacKenzie 2002	0 point	1 point	1 point	1 point	0 points	1 point	0 point	1 point	5 (Moderate)
Mishra 2018	0 point	0 point	1 point	1 point	0 points	0 point	0 point	1 point	3 (Low)
Nasrallah 2004	0 point	1 point	1 point	1 point	1 point	1 point	0 point	0 point	5 (Moderate)
Pearson 2011	0 point	1 point	1 point	1 point	2 points	0 point	1 point	0 point	6 (Moderate)
Schauberger 1994	0 point	1 point	1 point	1 point	1 point	1 point	0 point	1 point	6 (Moderate)
Singh 2012	0 point	1 point	1 point	0 point	2 points	1 point	1 point	1 point	7 (High)
Sunsaneevithayakul 2022	0 point	0 point	0 point	0 point	2 points	1 point	0 point	0 point	3 (Low)
Temesgen 2020	1 point	1 point	1 point	1 point	1 point	1 point	1 point	0 points	7 (High)
Tuffnell 2001	0 point	0 point	1 point	1 point	2 points	1 point	0 point	0 point	6 (Moderate)

^**#**^Quality score: 0–3 = high risk; 4–6 = moderate risk and 7–9 = low risk

**Table 3:** Quality assessment of included cross-sectional and retrospective studies

Study Identifier	Representativeness of sample	Sample size justification	Non-respondents	Exposure ascertainment	Comparability	Outcome assessment	Statistical test	Quality points^#^ (Grade)
Anuradha 2020	0 points	0 points	0 points	1 point	1 points	1 point	0 points	High (3)
Ayele 2021	0 points	0 points	0 points	0 points	1 points	1 point	1 point	High (3)
Boriboonhirunsarn 2016	1 point	0 points	0 points	0 points	2 points	1 point	1 point	5 (Moderate)
Hirani 2017	1 point	0 points	1 point	0 points	1 point	1 point	0 points	4 (Moderate)
Nakintu 2016	1 points	0 points	0 points	1 point1	2 points	1 point	1 point	6 (Moderate)
Tashfeen 2017	1 points	1 points	0 points	0 points	1 points	0 points	0 points	3 (High)
Thomas 2004	0 points	0 points	0 points	0 points	1 points	1 points	0 points	2 (High)

### DDT and apgar score < 7

**Fig. 2:**
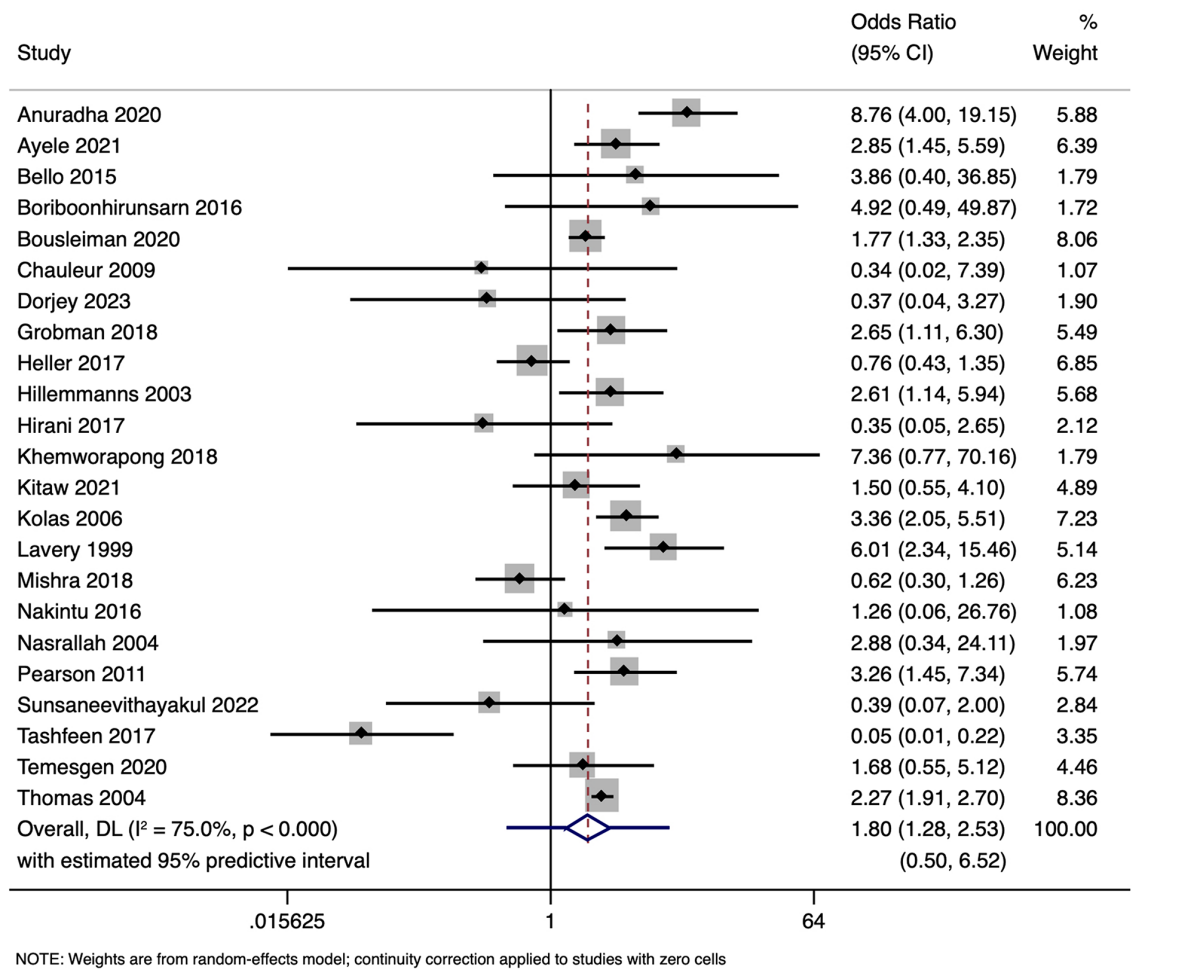
Forest plot showing the association between decision to delivery time (DDT) and Apgar score at 5 min

Apgar scores were reported in 23 studies involving 71,088 participants. Pooled analysis found a significant association between DDT < 30 min and an increased risk of Apgar score < 7 in neonates. The pooled OR was 1.80 (95% CI: 1.28–2.53) with prediction interval of 0.50 to 6.52 (Fig. 2). This shows that neonates born with DDT less than or equal to 30 min has 1.803 times higher odds of having Apgar score < 7, compared to neonates with DDT > 30 min.

**Fig. 3:**
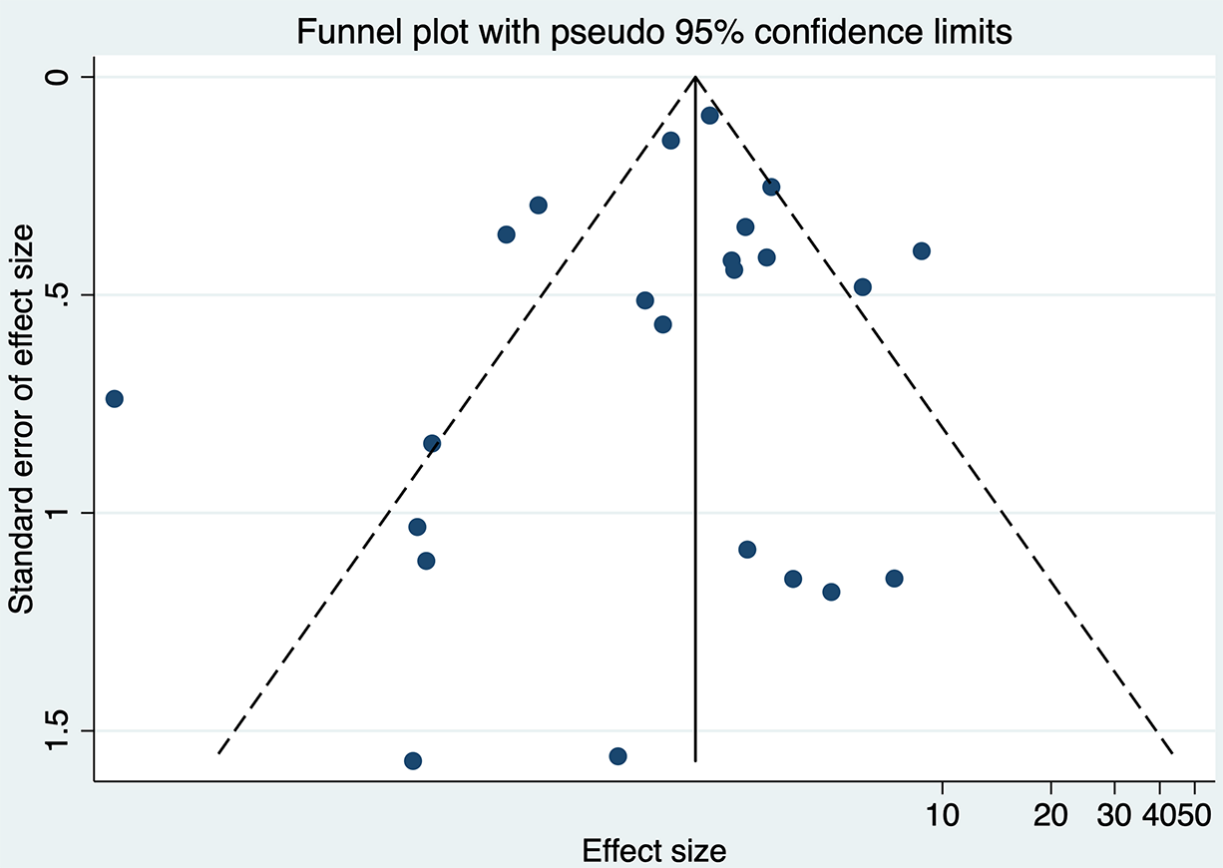
Funnel plot for Apgar score at 5 min

**Fig. 4:**
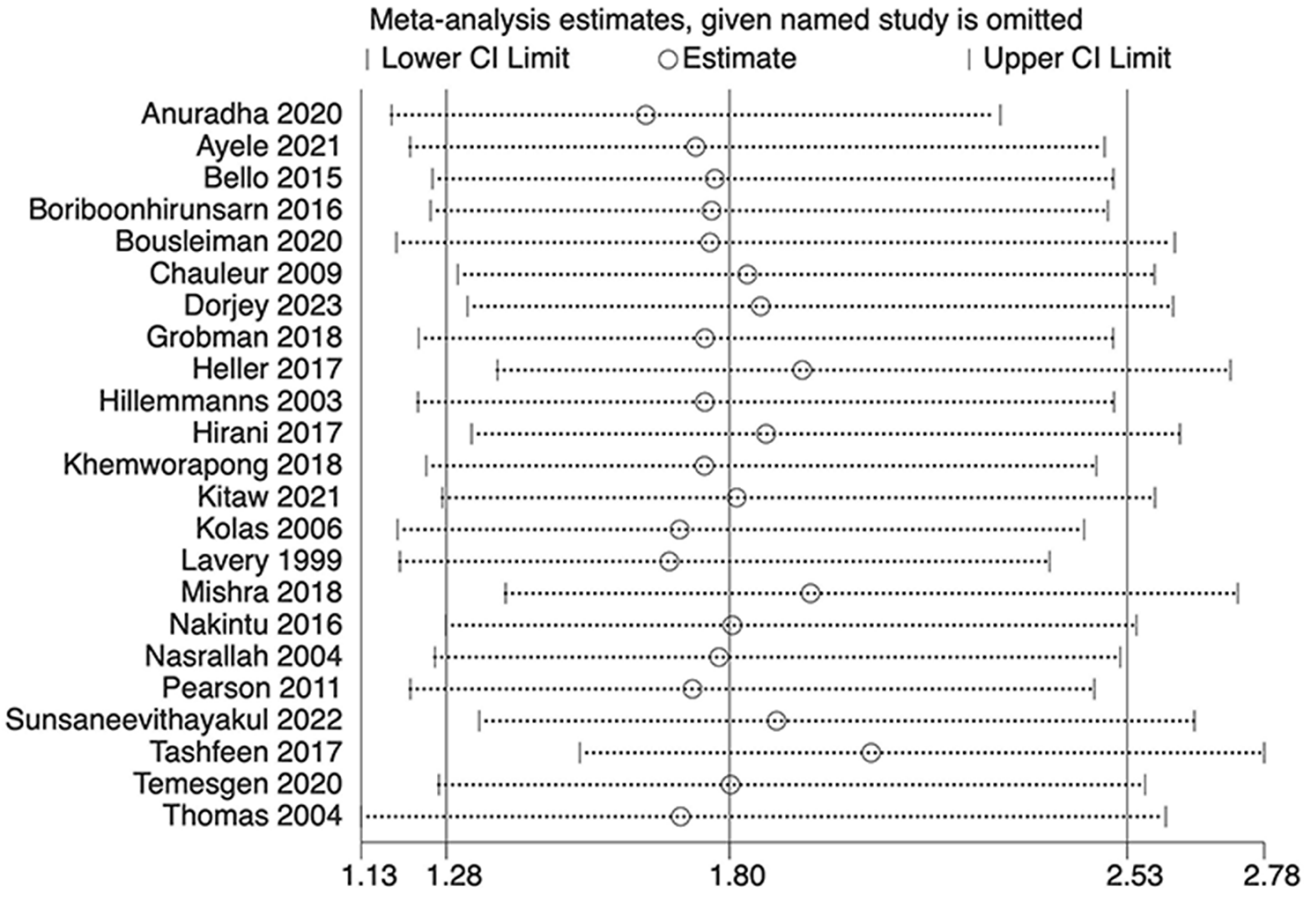
Sensitivity analysis plot for Apgar score at 5 min

The analysis indicated substantial heterogeneity (tau-squared = 0.35; I² = 75.0%, *p* < 0.001), suggesting variability in the study outcomes. Funnel plot (Fig. 3) showed a symmetrical plot indicating no publication bias, with Egger’s test further confirming it (*p* = 0.38). Sensitivity analysis (Fig. 4) did not reveal any single or small study effects contributing to heterogeneity. GRADE finding was reported to very low quality evidence because it was single downgraded due to inclusion of low quality studies, and again double downgraded due to presence of statistical heterogeneity, imprecision and indirectness (Table 4).

### DDT and umbilical cord pH < 7.1

**Fig. 5:**
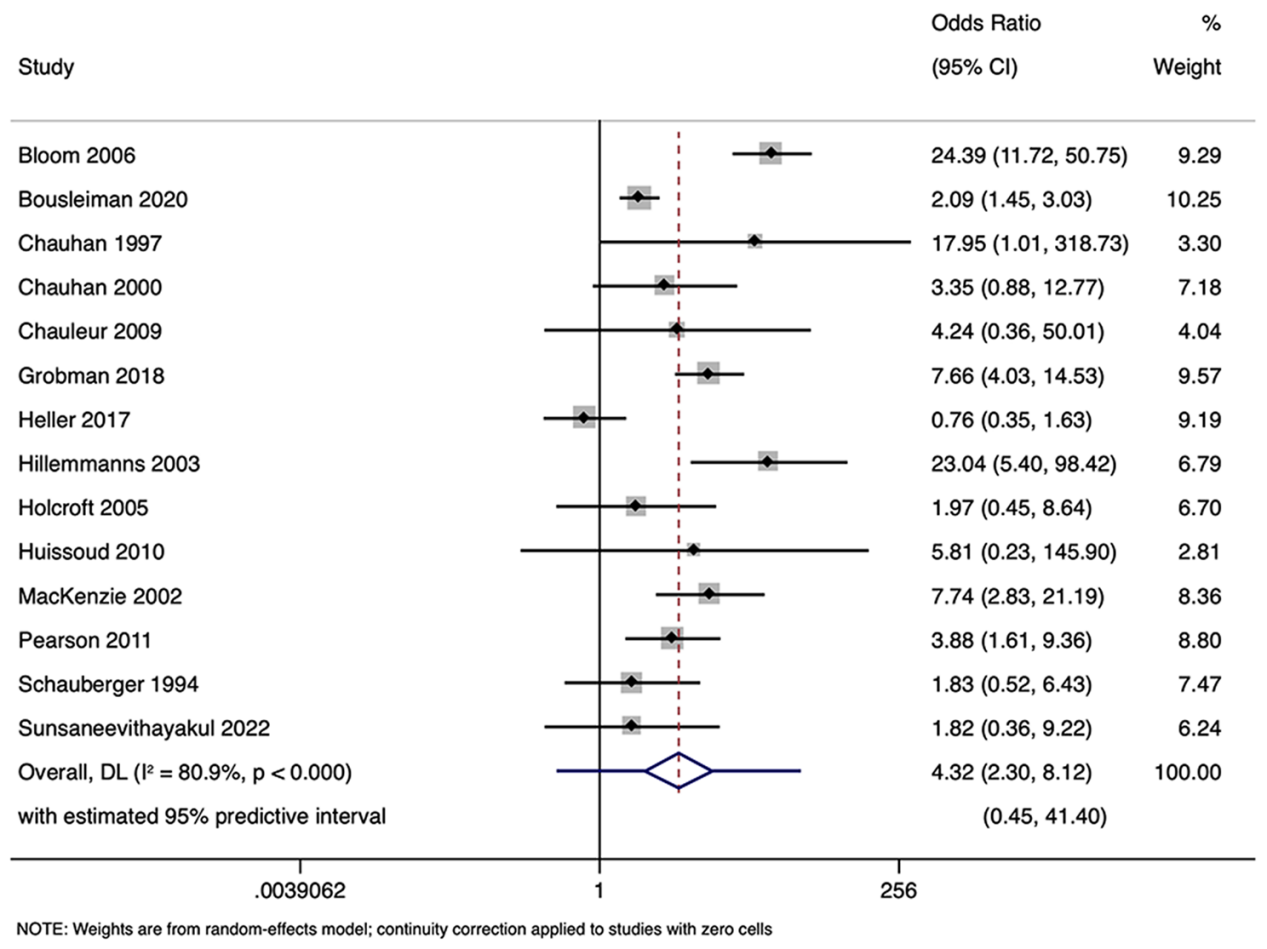
Forest plot showing the association between decision to delivery time (DDT) and umbilical cord pH

**Fig. 6:**
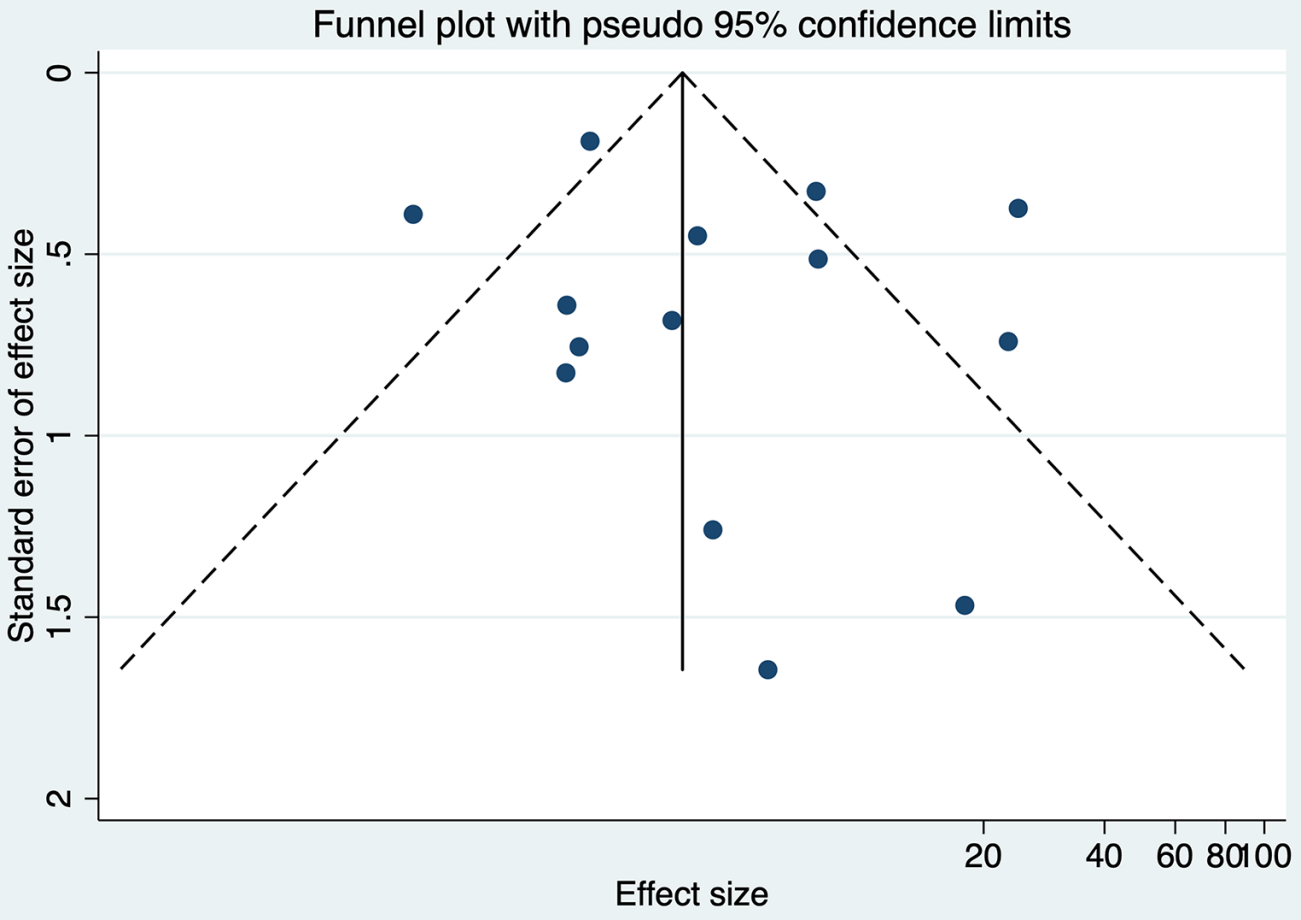
Funnel plot for umbilical cord pH

**Fig. 7:**
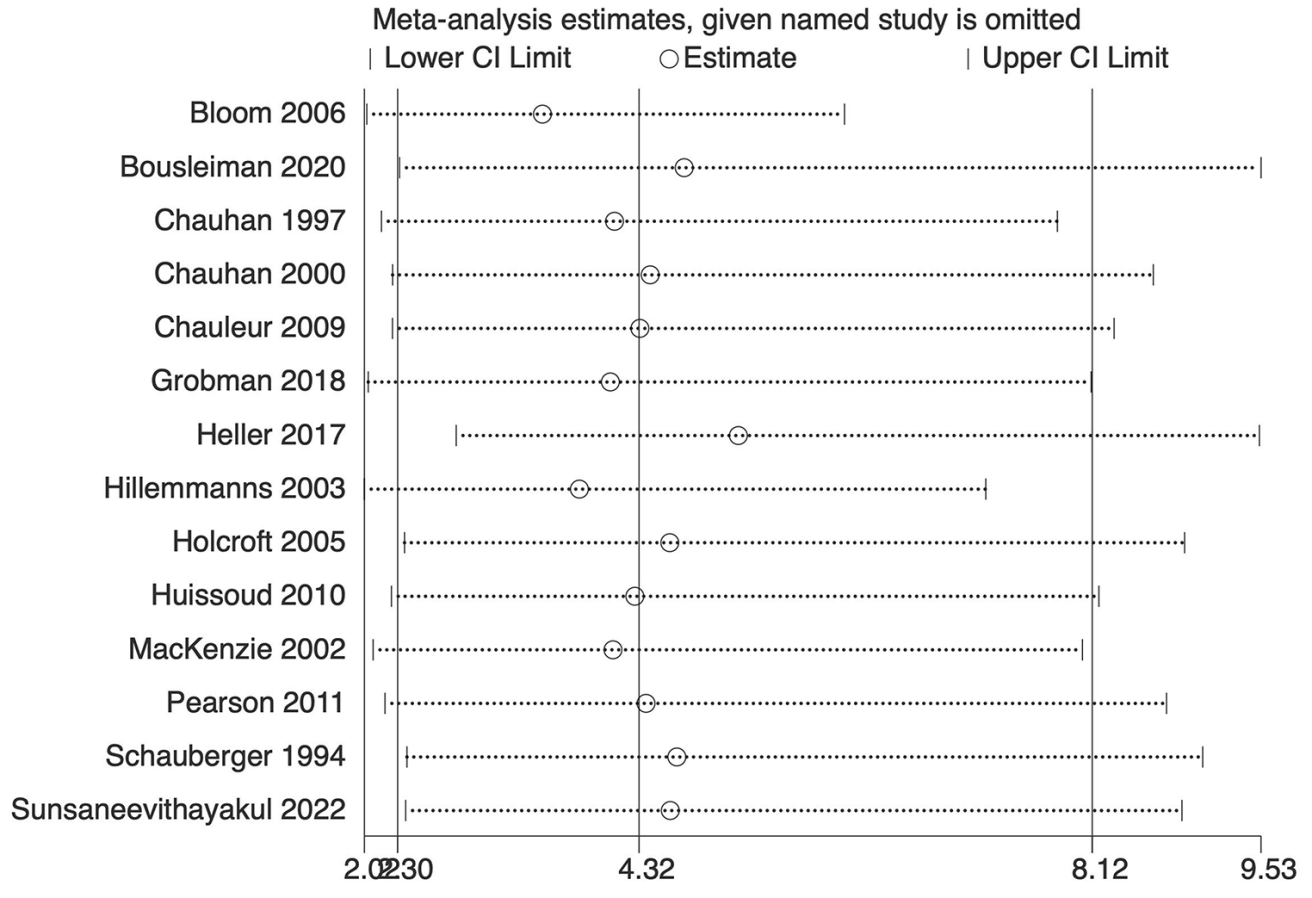
Sensitivity analysis plot for umbilical cord pH

Fourteen studies with a total of 48,234 participants reported umbilical cord pH measurements. There was a significant association between reduced DDT and increased odds of umbilical cord pH < 7.1. The pooled OR, was 4.32 (95% CI: 2.30–8.11) with prediction interval of 0.45 to 41.40 (Fig. 5). The analysis revealed considerable heterogeneity (tau-squared = 0.97; I² = 80.9%, *p* < 0.001), reflecting variability in the study results. Funnel plot (Fig. 6) showed a symmetrical plot indicating no publication bias, further confirmed by the Egger’s test (*p* = 0.42). Sensitivity analysis (Fig. 7) did not reveal any single or small study effects contributing to heterogeneity. GRADE finding was reported to very low quality evidence because of inclusion of low quality studies, presence of statistical heterogeneity and imprecise estimates (Table 4).

### DDT and NICU admission

**Fig. 8:**
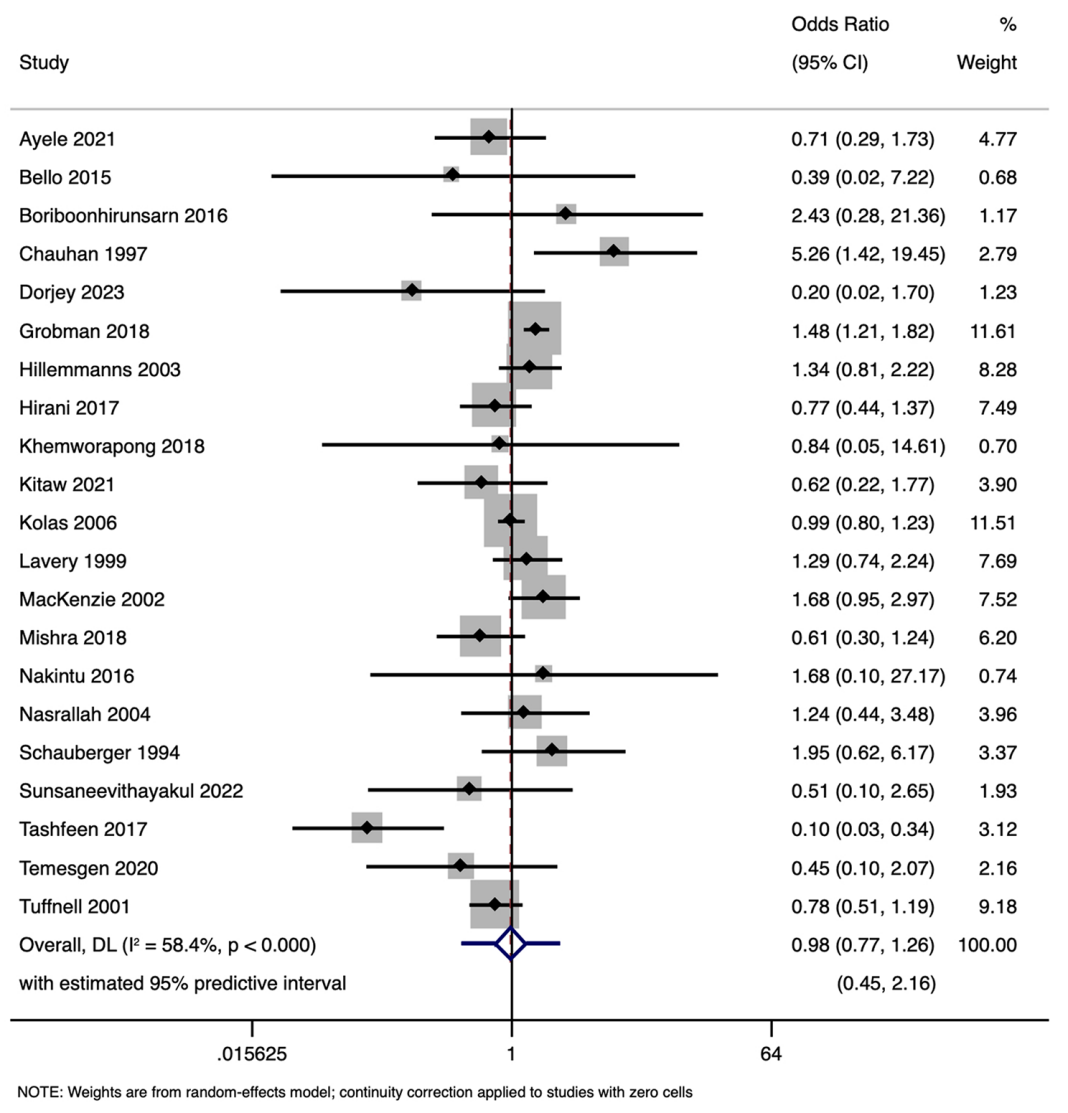
Forest plot showing the association between decision to delivery time (DDT) and neonatal intensive care unit admission

**Fig. 9:**
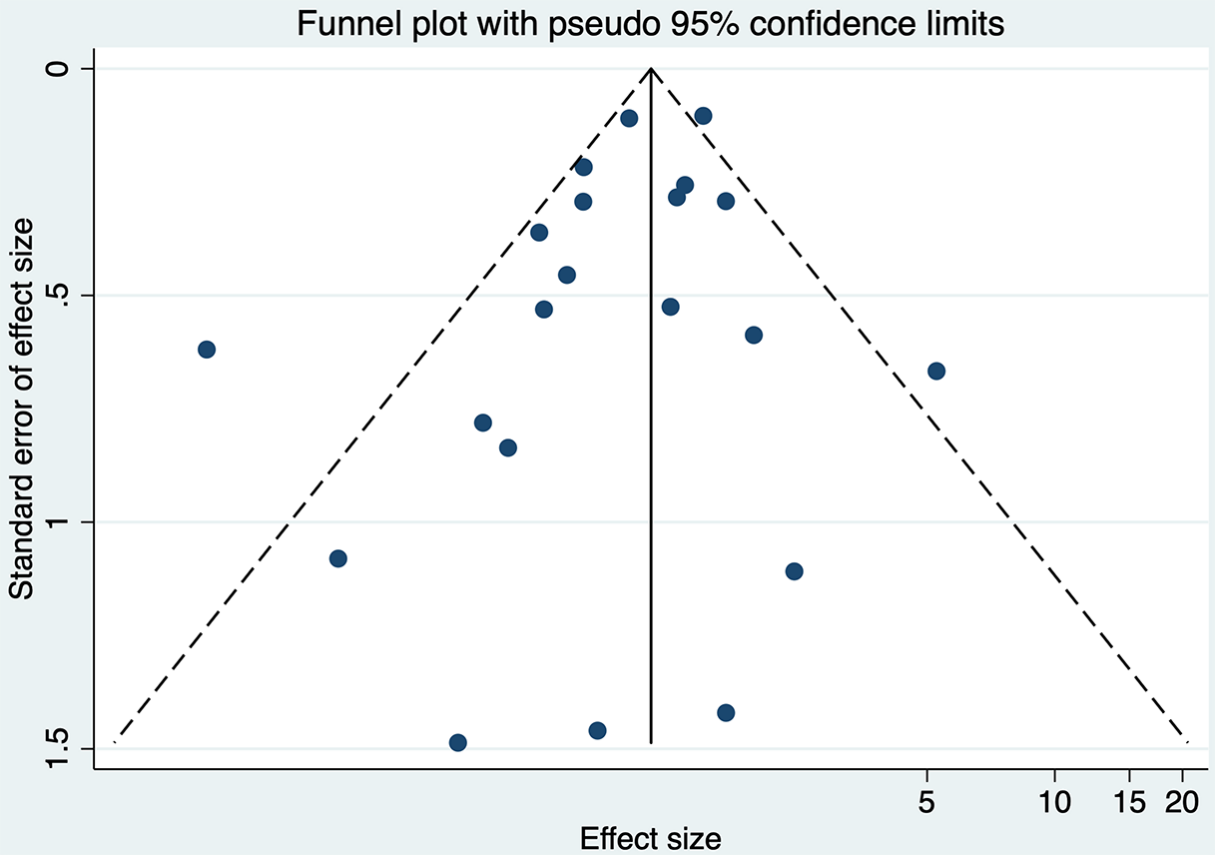
Funnel plot for neonatal intensive care unit admission

**Fig. 10:**
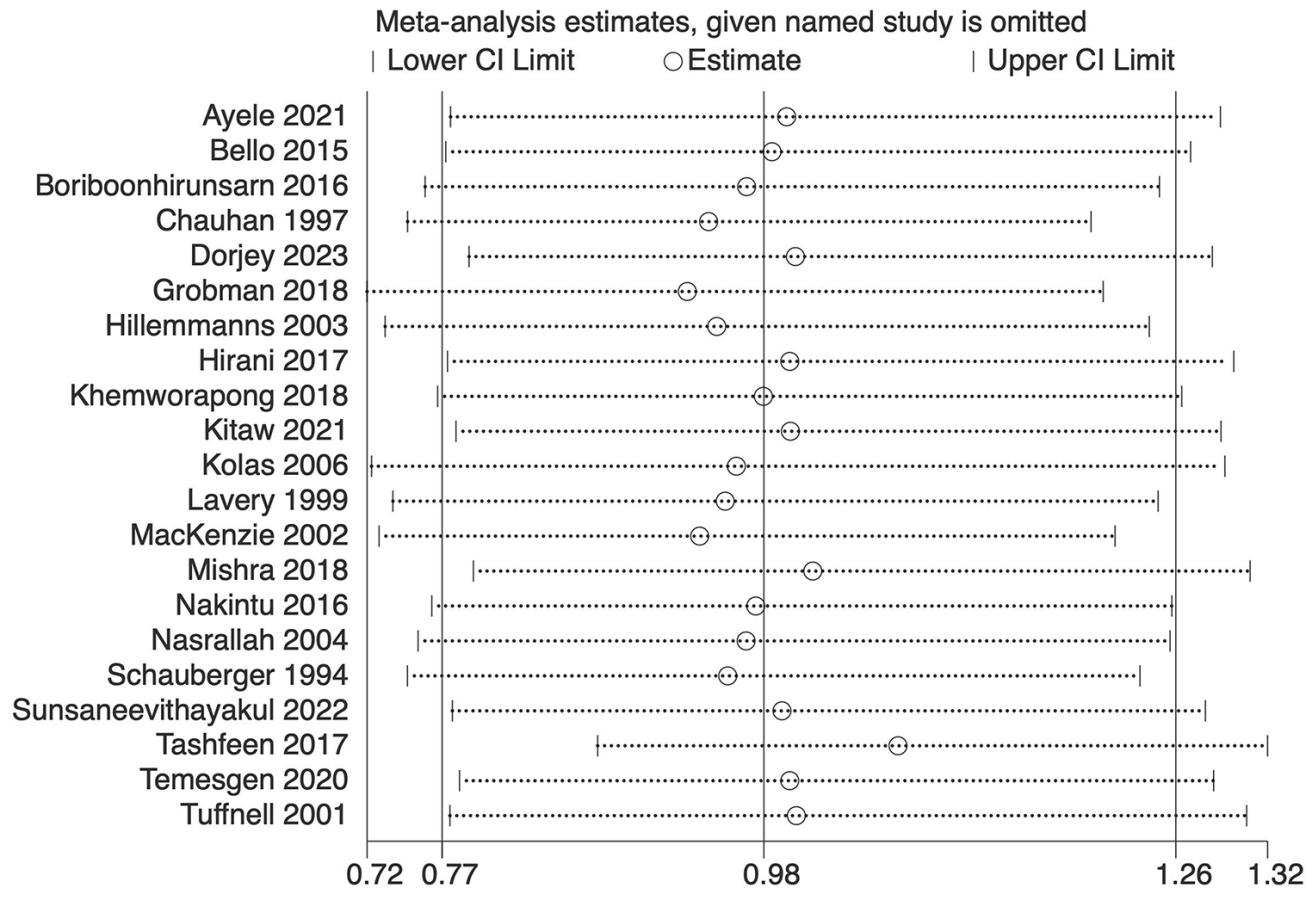
Sensitivity analysis plot for neonatal intensive care unit admission

A total of 21 studies with 10,697 participants have examined the relationship between extended DDT and the likelihood of NICU admission. The pooled analysis yielded an overall OR of 0.98 (95% CI: 0.77–1.26) with prediction interval of 0.45 to 2.16 (Fig. 8). The heterogeneity among the included studies was moderate (tau-squared = 0.13; I² = 58.4%, *p* < 0.001). This outcome suggests that prolonged or reduced DDT may not have a substantial impact on the rate of NICU admissions, although variations across the studies indicate a need for cautious interpretation of these findings. Funnel plot (Fig. 9) and Egger’s test did not detect publication bias (*p* = 0.17). Sensitivity analysis (Fig. 10) did not reveal any single or small study effects contributing to heterogeneity. GRADE finding was reported to very low quality evidence because of inclusion of low quality studies, presence of statistical heterogeneity and pooled estimates crossing the null value (Table 4).

**Table 4:** GRADE Findings of the study outcomes. Author(s): Shen et al. Question: Association between decision-to-delivery time and neonatal outcomes: A systematic review and meta-analysis

Certainty assessment							№ of patients		Effect		Certainty	Importance
№ of studies	Study design	Risk of bias	Inconsistency	Indirectness	Imprecision	Other considerations	DDT ≤30 min	DDT > 30 min	Relative (95% CI)	Absolute (95% CI)		
Apgar score < 7												
23	Observational studies	Serious^a^	very serious^b^	serious^f^	serious^g^	Publication bias undetected^j^	48,939	22,149	OR 1.80 (1.28 lower to 2.53 higher)	-	⨁◯◯◯ Very low	CRITICAL
**Umblical cord pH < 7.1**												
14	Observational studies	Serious^a^	very serious^c^	serious^f^	Very serious^h^	Publication bias undetected^j^	43,003	5231	OR 4.32 (2.30 lower to 8.11 higher)	-	⨁◯◯◯ Very low	CRITICAL
**NICU admission**												
21	Observational studies	Serious^a^	serious^d^	serious^f^	Very serious^i^	Publication bias undetected^j^	3537	7160	OR 0.98 (0.77 lower to 1.26 higher)	-	⨁◯◯◯ Very low	IMPORTANT
**Neonatal mortality**												
10	Observational studies	Serious^a^	not serious^e^	serious^f^	Very serious^i^	Publication bias undetected^j^	41,100	3790	OR 0.98 (0.56 lower to 1.71 higher)	-	⨁◯◯◯ Very low	IMPORTANT

**CI**: confidence interval; **OR**: odds ratio

^a^Single downgrade as there was Few studies had high risk of bias, while majority had moderate or low risk of bias

^b^Double downgrade as there was Substantial heterogeneity present (tau-squared = 0.35; I² = 75.0%)

^c^Double downgrade as there was Substantial heterogeneity present (tau-squared = 0.97; I² = 80.9%)

^d^Single downgrade as there was Moderate heterogeneity present (tau-squared = 0.13; I² = 58.4%)

^e^No downgrade as there was no heterogeneity present (tau-squared = 0; I² = 0.0%)

^f^Single downgrade as there was Indirectness in terms of study participants due to inclusion of studies conducted in both high income and low income countries

^g^Single downgrade as the sample size is high and CI of the final pooled estimate did not cross the null value. However the CI on both upper and lower end is beyond the recommended limit of 25% of the OR

^h^Double downgrade as the CI on both upper and lower end is way beyond the recommended limit of 25% of the OR

^i^Double downgrade as the CI of the final pooled estimate crossed the null value and upper and lower end is beyond the recommended limit of 25% of the OR

^j^No downgrade as there was no publication bias detected as the egger’s test was non-significant

### DDT and neonatal mortality

**Fig. 11:**
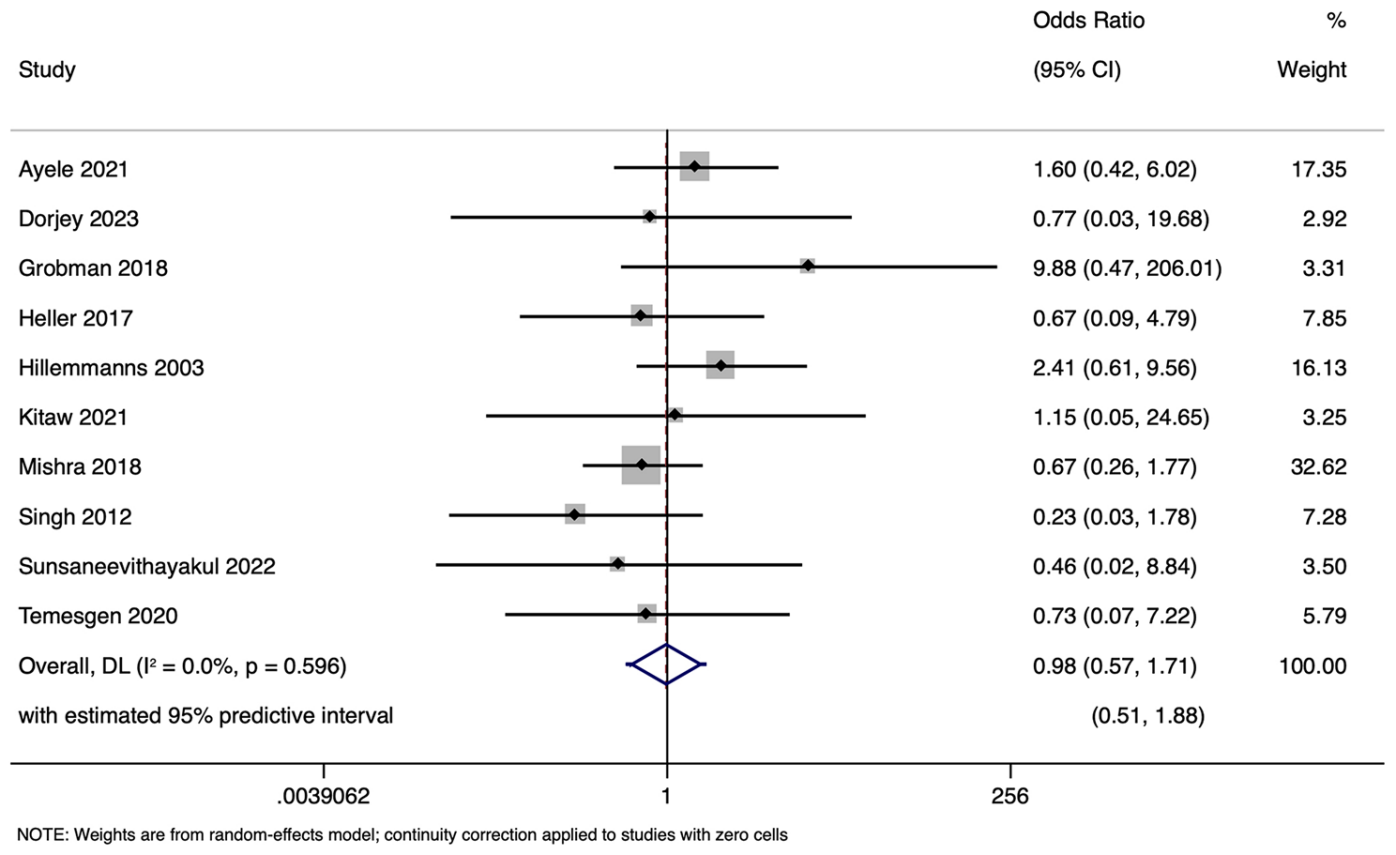
Forest plot showing the association between decision to delivery time (DDT) and neonatal mortality

**Fig. 12:**
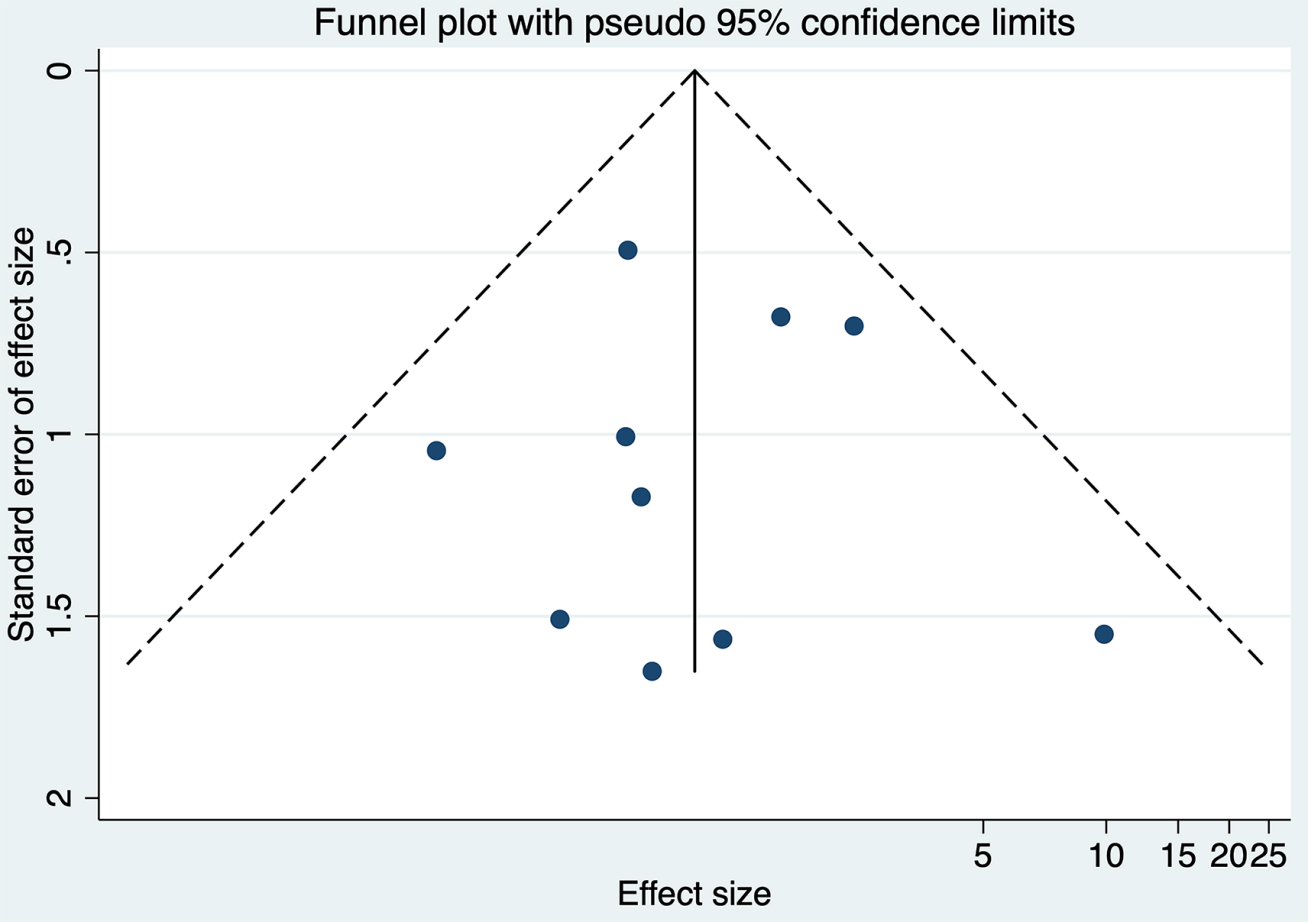
Funnel plot for neonatal mortality

**Fig. 13:**
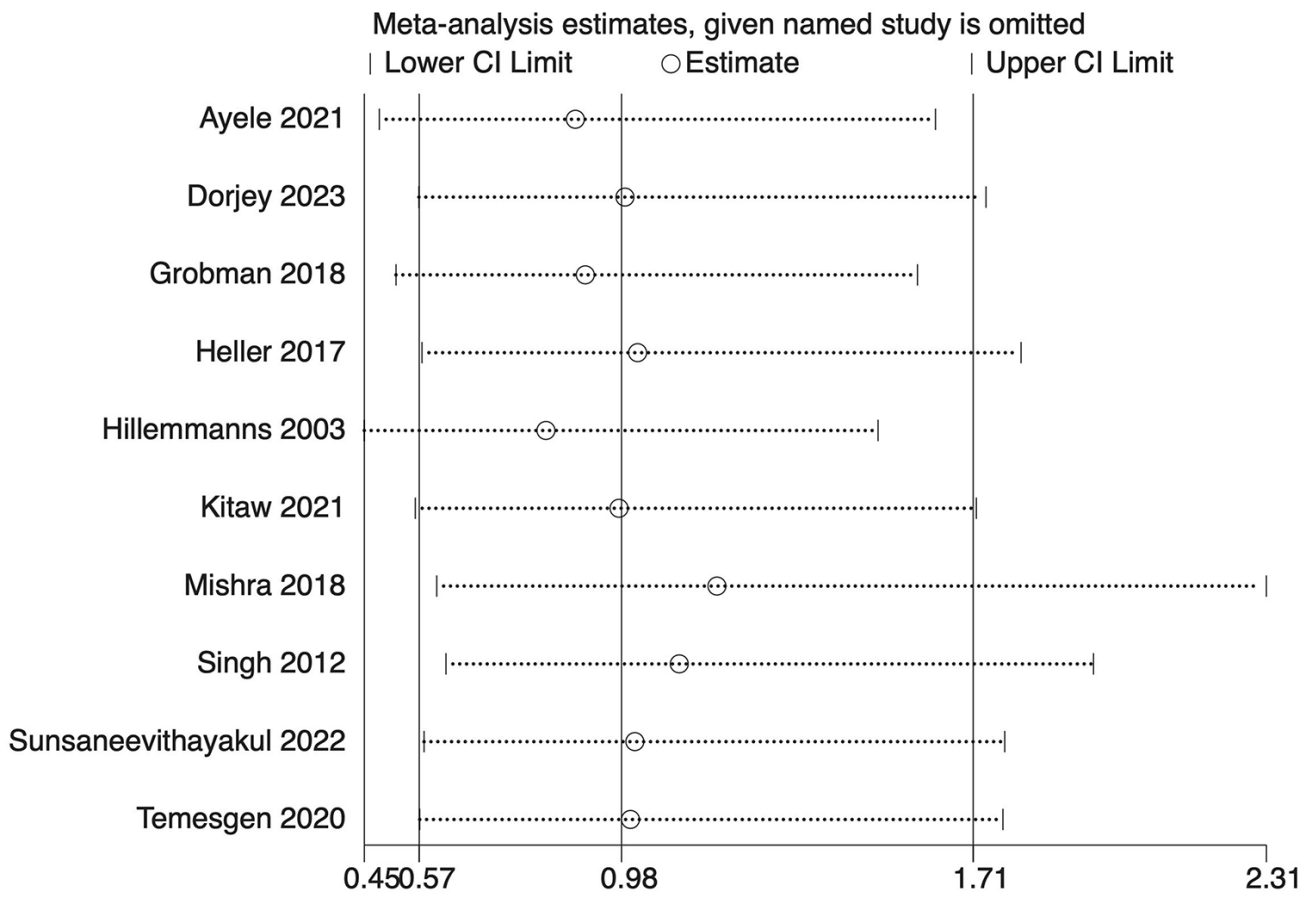
Sensitivity analysis plot for neonatal mortality

Ten studies encompassing 44,890 participants evaluated the association between DDT and neonatal mortality. The analysis produced an overall OR of 0.98 (95% CI: 0.56–1.71) with prediction interval of 0.51 to 1.88 (Fig. 11). Notably, the heterogeneity among the studies was negligible (tau-squared = 0; I² = 0.0%, *p* = 0.596). This finding suggests that the length of decision-to-delivery time may not have a significant impact on neonatal mortality, based on the currently available data. Funnel plot (Fig. 12) showed a symmetrical plot indicating no publication bias, as confirmed by the results of the Egger’s test (*p* = 0.91). Sensitivity analysis (Fig. 13) did not reveal any single or small study effects contributing to heterogeneity. GRADE finding was reported to very low quality evidence because of inclusion of low quality studies and pooled estimates crossing the null value. However, there was absence of statistical heterogeneity, publication bias and directness in evidence (Table 4).

## Discussion

Our review revealed significant associations between DDT below 30 min, and Apgar scores < 7 and acidosis. We found no significant association of DDT with mortality and NICU admission rates. Our findings provide critical insights into the time-sensitive nature of obstetric emergencies and their impact on neonatal health.

Our findings are consistent with earlier studies indicating that while in some cases caesarean section necessitate a shorter DDT, the majority of neonates may be safely delivered within a longer interval of time [11, 45, 46]. The lack of a significant association with NICU admissions and neonatal mortality in our study differ diverge from previous reports that showed a direct correlation between delayed delivery and elevated risks of these outcomes. This discrepancy could be attributed to variations in study methodologies, and population demographics. Additionally, there may be possible differences in healthcare infrastructure and emergency response protocols and the varying levels of resources available in high versus low-income countries [11]. Advancements in neonatal care, particularly in high-resource settings, might have mitigated the impact of delivery time on outcomes like NICU admission and neonatal mortality, a factor less explored in earlier studies.

This notable discrepancy with previous reviews, particularly in the context of high-income versus low-income countries is concerning. Prior studies have predominantly focused on high-income settings, where the infrastructure and resources available for obstetric care are typically more advanced. Indeed, some previous reviews have explicitly excluded low-income countries from their analyses, based on the assumption that a 30-minute interval is not achievable in these settings.

However, our findings suggest that striving for a 30-minute decision-to-delivery time is crucial, irrespective of the country’s income level. This underscores the importance of including diverse healthcare settings in future research to understand the universal applicability and benefits of rapid emergency obstetric care. Ensuring that all countries, regardless of income level, are represented in such studies is essential to developing global guidelines that are both realistic and effective in improving neonatal outcomes.

The significant association between reduced DDT and increased risk of Apgar scores < 7 and low umbilical cord pH may be linked to the rapid progression of fetal distress in emergency scenarios [47]. Additionally, the confounding effect of the severity of maternal and fetal conditions leading to emergency caesarean sections cannot be overlooked [11]. It is plausible that cases requiring a DDT of less than 30 min were inherently more severe, thus predisposing neonates to adverse outcomes irrespective of the delivery time. This factor might have significantly influenced the increased odds of lower Apgar scores and acidosis in neonates delivered within a shorter DDT. Therefore, while a shorter DDT is crucial, it may also be a marker of more severe underlying complications, which in themselves contribute to outcomes like low Apgar scores and acidosis. The absence of a similar trend in NICU admissions and neonatal mortality could be influenced by advancements in neonatal care, which mitigate the impact of delivery delays on these outcomes.

Our findings underscore the need for a more nuanced approach in clinical decision-making.

It is imperative for clinicians to balance the urgency of delivery with the underlying clinical context. Rapid decision-making and action are paramount in emergency caesarean deliveries to mitigate risks of low Apgar scores and acidosis. However, this urgency must be balanced with a thorough assessment of the underlying clinical conditions. Our study suggests that the severity of maternal and fetal conditions may play a critical role in neonatal outcomes. Therefore, interventions should be tailored to address these specific conditions, beyond the sole focus on reducing DDT.

In addition, our study acknowledges the relevance of Lucas categories in assessing neonatal outcomes. The evolution of obstetric guidelines, particularly in relation to these categories, underscores the dynamic nature of this field. However, there remains a gap in understanding how these guidelines translate across different income settings. Our findings prompt a call for more research into the implementation and effectiveness of these guidelines, especially in low-income countries where resources and healthcare infrastructure may significantly impact DDT and its associated outcomes.

The strength of our study lies in its comprehensive dataset, encompassing a large cohort from diverse demographics, which enhances the reliability and applicability of our findings. Our rigorous methodology and the absence of publication bias further strengthen the credibility of our results.

However, our study has certain limitations. Tere is a risk of potential selection biases due to the focus on English-language studies, and the inherent variability in study designs among the included studies. These factors may limit the generalizability of our conclusions and suggest the need for further research in more diverse linguistic and cultural settings.

Future research should aim to identify the factors contributing to the observed discrepancies in outcomes associated with DDT. Longitudinal studies examining the long-term impacts of DDT on neonatal health, and studies in diverse healthcare settings, are needed. Additionally, studies should explore the role of healthcare system efficiency and obstetric care protocols in modifying the impact of DDT on neonatal outcomes. Future studies should specifically focus on disentangling the effects of DDT from the confounding influence of the severity of maternal and fetal conditions. Investigating these factors separately could provide more clarity on the direct impact of DDT on neonatal outcomes. Additionally, multi-centric studies encompassing diverse healthcare settings could offer more generalizable insights.

## Data Availability

The datasets generated and analysed during the current study are available in the PubMed, Scopus, Cochrane Library, and Google Scholar.
